# Exploring the Relationship Between Cooking and Food Skills and Eating Competence Among Brazilian Adults

**DOI:** 10.3390/nu16233980

**Published:** 2024-11-21

**Authors:** Maísa Lins, Eduardo Yoshio Nakano, João Rafael Queiroz Soares, Fabiana Lopes Nalon de Queiroz, Raquel Braz Assunção Botelho, António Raposo, Renata Puppin Zandonadi

**Affiliations:** 1 Department of Nutrition, Faculty of Health Sciences, Campus Universitário Darcy Ribeiro, University of Brasília, Brasilia 70910-900, Brazil; maiflins@gmail.com (M.L.); fabinalon@hotmail.com (F.L.N.d.Q.); raquelbotelho@unb.br (R.B.A.B.); 2 Department of Statistics, Campus Universitário Darcy Ribeiro, University of Brasília, Brasilia 70910-900, Brazil; nakano@unb.br; 3 Department of Medicine, Faculty of Education and Health Sciences, Campus Asa Norte, Centro Universitário de Brasília (CEUB), Brasilia 70790-075, Brazil; jrafaqs@gmail.com; 4 CBIOS (Research Center for Biosciences and Health Technologies), Universidade Lusófona de Humanidades e Tecnologias, Campo Grande 376, 1749-024 Lisboa, Portugal

**Keywords:** Brazilian adults, cooking skills, eating competence, food preparation, health, nutrition

## Abstract

Background: Cooking skills (CSs) and food skills (FSs) are essential in promoting healthier eating habits. Eating competence (EC) encompasses an individual’s self-regulation, enjoyment, and positive attitude toward food, contributing to their overall well-being. However, no research has explored the relationship between CSs, FSs, and EC, particularly within the Brazilian context. Objective: This study aimed to assess the association between EC and CFSs among Brazilian adults. Methods: A cross-sectional survey was conducted among 1266 Brazilian adults using two validated instruments: the Cooking and Food Skill Confidence Questionnaire (CFS) and the Brazilian version of the Satter Eating Competence Inventory (ecSI2.0™BR). Pearson’s and Spearman’s correlations were used to analyze the relationships between CSs, FSs, and EC. Results: A significant positive correlation was found between higher levels of CSs and the overall EC (r = 0.417, *p* < 0.001), with complex cooking tasks showing stronger associations with EC. Contextual skills within EC exhibited the highest correlation with FSs (r = 0.487). Basic tasks, such as microwaving food, showed weaker associations with EC. Conclusions: The findings suggest that enhancing cooking and food skills may support greater eating competence, promoting healthier eating behaviors. Public health programs should consider integrating cooking skill development to foster better dietary outcomes and improve individual well-being.

## 1. Introduction

Food consumption outside the home is an increasingly common practice in modern society. People choose to eat at restaurants, fast food, and other food services due to convenience, personal preference, lack of time for commuting and meal preparation, or a lack of cooking and food skills (CSs and FSs) [1,2]. Several arguments are used to justify the decline in the habit of preparing meals at home, including the convenience and ease of acquiring ready-to-eat products, the lack of time for menu planning, grocery shopping, and food preparation, not knowing how to cook or not enjoying it, the pleasure of dining out, and considering cooking to take too much effort [1,3,4,5].

It is known that involvement in food preparation allows individuals greater control over the ingredients used, enabling them to choose fresher, healthier options with lower levels of saturated fats, sugars, and sodium [6]. Thus, skills in selecting ingredients, preparing meals (cooking skills), and the willingness to cook may play a crucial role in preventing non-communicable chronic diseases and overweight, as they promote the preparation and consumption of healthier meals [7,8].

Brazilian culture is marked by a strong food tradition, where home cooking and family meal consumption are deeply valued practices passed through generations. Cooking at home is often seen as an expression of care and affection, reflecting not only the importance of a balanced diet but also the role of food in social cohesion and family dynamics [9]. Studies show that this cultural context can play a significant role in developing cooking and food skills, as culinary learning is primarily influenced by family interaction and an emphasis on fresh, local ingredients [5,10].

Additionally, the Brazilian Dietary Guidelines highlight the importance of eating regularly and mindfully, in appropriate environments, and, whenever possible, in the company of others, which reinforces the connection between culture and food and encourages practices which enhance eating competence by fostering a more mindful and enjoyable relationship with food [9]. These cultural aspects shape cooking and food skills and attitudes and behaviors toward food, influencing how Brazilians approach meal preparation and consumption. Therefore, in a context where home-cooked meals are encouraged and food traditions are deeply rooted, EC is likely to be promoted, fostering healthier food choices and a balanced relationship with eating [11].

By knowing basic cooking techniques, such as cutting, cooking, and combining ingredients, individuals gain more autonomy in creating healthy meals and adapting them to their preferences and dietary needs [9]. In this context, CSs and FSs have become a focus of study for evaluating healthy eating practices in various countries [12,13,14,15,16,17].

Possessing and developing these skills is essential for maintaining a healthy diet, as it allows individuals to prepare appropriate meals at home using fresh and wholesome ingredients, as recommended by the Brazilian Dietary Guidelines [9]. CSs and FSs are also related to understanding nutritional principles and the importance of a balanced diet. Hence, acquiring CSs and FSs is intrinsically linked to eating competence, contributing to conscious, flavorful, and healthy choices [18].

Eating competence (EC) is an approach that recognizes eating as a complex process, encompassing learned behaviors, social expectations, cultural preferences, and attitudes and emotions related to food [19]. EC is composed of four components: eating attitudes (enjoyment of food in satisfying amounts), food acceptance (interest in food variety and experimentation), internal regulation (awareness of hunger and satiety signals), and contextual skills (managing the eating environment and planning regular meals) [20]. “Competent eaters” can comfortably and positively select what and how much to eat, internalizing hunger and satiety cues in association with the context and situation [11,20].

Research has shown that EC correlates with psychological and behavioral aspects, including higher body weight satisfaction and reduced disordered eating behaviors [21]. Moreover, Lohse et al. (2012) suggest that EC is closely associated with an increased consumption of fruits and vegetables, as well as the development of more advanced skills in food handling and the self-management of one’s diet [22].

Cooking and food skills provide a foundation for developing EC, as defined by Satter (2007), which includes an individual’s ability to make informed food choices, enjoy eating, and regulate food intake to promote nutritional adequacy and overall well-being [19]. CSs, such as the mechanical processes involved in meal preparation and the cognitive understanding of food transformation, are essential for preparing meals at home. Similarly, food skills, which involve selecting ingredients, meal planning, budgeting, and ensuring nutritional balance, contribute to an individual’s ability to provide satisfying and age-appropriate meals [13,23,24].

Together, these skills align closely with the principles of EC, encouraging a positive relationship with food, meal planning, and an ability to manage eating in a structured yet flexible way. Since CSs can promote better autonomy in food selection and a deeper appreciation for cooking, resulting in a more positive relationship with food and overall healthier eating habits, it is hypothesized that the higher an individual’s cooking skills, the more eating-competent this person will be.

Public health interventions targeting cooking and food skills aim to improve dietary habits by building confidence and practical skills for preparing healthy meals. Programs like Cooking Matters in the U.S. and Jamie’s Ministry of Food in Australia focus on hands-on education in meal planning, budgeting, and cooking techniques, showing success in increasing vegetable intake, reducing food insecurity, and encouraging home-cooked meals [25,26].

In Brazil, the Brazilian Dietary Guidelines promote traditional cooking and discourage unhealthy industrialized foods, emphasizing mindful eating and the social aspects of meals [9]. Such initiatives demonstrate the role of cooking skills in supporting healthier food choices and improving long-term health outcomes [2].

Some research correlates EC with healthy habits, the availability of fruits and vegetables at home, and individuals’ quality of life; others investigate cooking and food skills concerning eating behaviors and quality of life [6,13,27,28,29,30]. EC, CSs, and FSs are evaluated internationally with similar variables and comparisons, but no studies have been identified in the literature evaluating the association of CSs and FSs with EC.

Given this context, there is a gap in the literature regarding the relationship between FSs, CSs, and EC. This study hypothesizes a direct and positive relationship between FSs, CSs, and EC, where individuals with more developed cooking and food skills are expected to demonstrate higher levels of eating competence. Grounded in the findings that higher skill levels correlate with improved dietary choices, greater food autonomy, and increased confidence in meal preparation, our research seeks to understand whether Brazilian adults with greater cooking and food skills are more eating-competent.

Therefore, this study aims to explore whether there is a relationship between cooking and food skills and EC among Brazilian adults, to support the promotion of healthier eating habits, efficient public health actions, and, consequently, an improved quality of life.

## 2. Materials and Methods

### 2.1. Study Characterization and Ethical Aspects

This cross-sectional study used a non-probabilistic convenience sample design to explore the relationship between cooking skills and eating competence among Brazilian adults. The research was divided into three steps: (i) selection of the instruments; (ii) validation of the chosen instrument—“Cooking and Food Skill Confidence Questionnaire” in its Brazilian Portuguese online version—to evaluate cooking skills; and (iii) nationwide survey using (1) socio-demographic data, (2) the Cooking and Food Skill Confidence Questionnaire in its Brazilian version (CFSCQ-BR), and (3) the Satter Eating Competence Inventory in Brazilian Portuguese (ecSI2.0™BR) [11].

This study was conducted in accordance with the Declaration of Helsinki and the Research Ethics Committees’ CEP (3.769.157 and CAAE: 24415819.2.0000.8101) and approved by the NEEDs Center, holder of the copyright for using the instrument that assesses eating competence (ecSI2.0™BR).

### 2.2. Selection and Characteristics of the Instruments Selected for This Research

Socio-demographic data were collected through a structured questionnaire, which captured key variables, including age, gender, ethnicity, place of residence in Brazil, educational level, monthly household income, and the number of children and adults living in the household. These factors provided a detailed profile of the participants, offering an important context for understanding how cooking skills, food skills, and eating competence are developed and practiced within different demographic settings.

Cooking skills were evaluated using the Cooking and Food Skill Confidence Questionnaire instrument, first published by Lavelle et al. (2017) and translated into Brazilian Portuguese [12,31] (Supplementary Materials—Tables S1 and S2). This instrument was chosen based on an extensive literature review previously registered with the Open Science Framework (<osf.io/g86wd>) to ensure the identification of ongoing reviews and avoid an unnecessary duplication of research. The instrument consists of 33 items assessed on an 8-point Likert scale (0 to 7), with the final score calculated as the mean confidence rating for each respondent. This score is derived exclusively from individuals who report possessing the skill and provide a self-assessed intensity rating. Responses marked as “zero” were excluded from the analysis, as they indicated non-use of the skill, making it impossible to evaluate their level of proficiency. Cooking and food skill (CFS) confidence is measured for each domain (cook and food skills isolated); higher scores indicate greater cooking skills [31].

Since the CFS original instrument uses the mean score for response analysis on a 7-point scale, disregarding the zero rating as previously mentioned, we chose to categorize individuals with a mean score of ≥5 as “skilled” in both domains (CF and FS) and the overall classification (CFS). People with a score below one-third of the total possible points were supposed to lack good cooking and food skills. Therefore, the cutoff point was set to 5 (those with a mean score above the second tercile), aligning with the cutoff used in the EC instrument. Eating competence was assessed using the Brazilian version of the Satter Eating Competence Inventory (ecSI2.0™BR), the only one which evaluates EC and has been translated and validated for the Brazilian population [28,32]. This questionnaire is available upon request on the NEEDs Center website and requires authorization for use. The ecSI2.0™BR is composed of 16 items scored on a 5-point Likert scale (Always = 3 to Rarely/Never = 0). The score is defined as the sum of the responses for each of these items. Thus, the punctuation varies from 0 to 48 [33]. To be considered a competent eater, the person has to score a minimum of 32, and higher scores indicate greater EC [20,33].

### 2.3. Validation of the Brazilian Online Version of the Cooking and Food Skill Confidence Questionnaire (CFSCQ-BR)

The Cooking and Food Skill Confidence Questionnaire was translated into Brazilian Portuguese in a previous study, following the Beaton and collaborators’ model: translation, translation synthesis, back-translation, expert committee, and pre-test to be applied in person [31,34]. The instrument’s semantic, idiomatic, cultural, and conceptual equivalences were analyzed, obtaining a cutoff point equal to or greater than 80% of consensus among the expert committee [31]. However, the instrument was only validated for in-person research and not online applications.

Therefore, to be able to use it in this research, it was necessary to validate the instrument online. In this sense, the CFSCQ-BR’s reproducibility (reliability) and internal consistency were evaluated in the present study through a convenience non-probabilistic sample. A total of 60 individuals were invited to take part in this stage. However, only 42 agreed to participate and were divided into 2 groups (Group 1 answered online–online versions; and Group 2 answered printed–online versions). For the reproducibility analysis, each participant completed the instrument at two different time points, with a minimum interval of 48 h and a maximum of 15 days. Of the 42 participants in the first round, 41 completed both rounds of responses.

The participants had not been previously informed that they would need to complete the questionnaire twice. One group (n = 21) responded to the online version of the questionnaire, and, after a minimum of 48 h, the group was instructed to repeat it (also in the online version). The other group (n = 20) completed the printed version of the questionnaire first and, after the same minimum interval, was asked to provide their second response using the online format.

The test–retest agreement was evaluated using the intraclass correlation coefficient (ICC), with ICC values greater than 0.75 considered excellent. Additionally, the instrument’s internal consistency was assessed using Cronbach’s Alpha, with values greater than 0.7 indicating good consistency.

### 2.4. Nationwide Survey

After confirming CFSCQ-BR’s reproducibility (reliability) and internal consistency for online application, the survey, comprising socio-demographic data, CFSCQ-BR, and ecSI2.0™BR [11], was inserted in the Google Forms platform for data collection.

Before starting the survey, participants were provided with an informed consent form explaining the purpose of the study, potential risks, and the confidentiality of their responses. Only those who agreed to participate by selecting the option “I have read and accept the Informed Consent Form” proceeded to answer the questionnaire.

The nationwide application consisted of a cross-sectional study conducted with a non-probabilistic convenience sample, recruited using the snowball recruitment method online because it reduced participant recruitment time and study costs while allowing for a larger sample size. This method is employed when reaching individuals with the desired characteristics is challenging. In snowball sampling, known participants recruit future participants among their acquaintances, creating a referral chain. This process continues until the sample reaches a sufficient size and data saturation is achieved [35].

The recruitment process predominantly took place online, using known networks and distributing the study link through various platforms, including social media, communication platforms, and emails.

### 2.5. Statistical Analysis

The scores for both the CFSCQ-BR and ecSI2.0™BR instruments, including their respective domains, were described using means and standard deviations. The same was applied to other quantitative variables in the study. Categorical variables were presented using absolute and relative frequencies.

Independent Student *t*-tests or an analysis of variance (ANOVA) followed by Tukey’s post hoc test were used to compare the CFSCQ-BR scores and ecSI2.0™BR scores with variables of interest. Comparisons between cooking and food skills were performed by paired Student *t*-test. Data normality was verified using the Kolmogorov–Smirnov test. Comparisons involving categorical variables were performed using Pearson’s Chi-square test, and the association between the Cooking and Food Skill Confidence Questionnaire and ecSI2.0™BR scores was measured using Pearson’s or Spearman’s correlation coefficient. All tests were two-tailed, with a significance level set to 5%. Statistical analyses were conducted using IBM SPSS Statistics for Windows (Version 22, IBM Corp., Armonk, NY, USA).

## 3. Results

**Figure 1:**
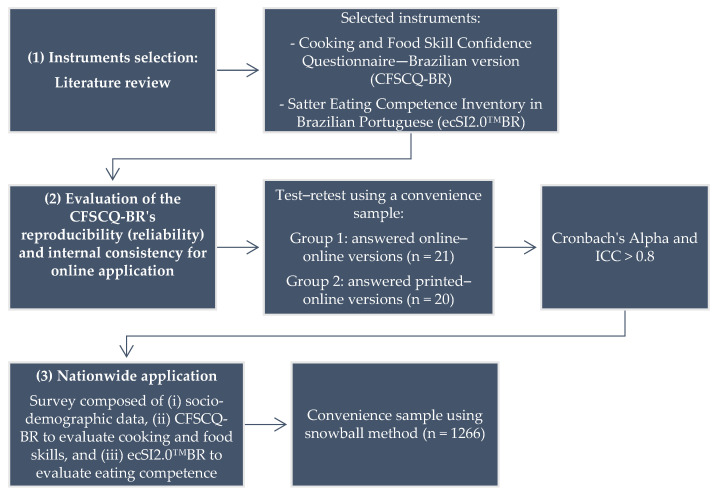
Flowchart of the steps for questionnaire selection and the evaluation of the Brazilian population, correlating CFSCQ-BR and ecSI2.0™ BR.

Following the comprehensive methodological approach detailed in Figure 1, the results of this study reveal key insights into the relationship between CFSs and EC among Brazilian adults.

### 3.1. Findings from the Validation of the Brazilian Online Version of the Cooking and Food Skill Confidence Questionnaire (CFSCQ-BR)

The validation sample was selected through random convenience sampling, comprising 41 individuals divided into 2 groups: 21 in Group 1 and 20 in Group 2. In both groups, the majority were female (Group 1: 15 participants, 71.43%; Group 2: 11 participants, 55.00%), as detailed in the Supplementary Materials (Table S3).

The participants were categorized into three age groups. In Group 1, the largest age group ranged from 21 to 30 years (n = 11, 52.38%), followed by 31 to 48 years (n = 7, 33.33%), and 61 to 73 years (n = 3, 14.29%). Group 2 had a similar distribution, with most participants aged 21 to 30 years (n = 12, 60.00%), followed by 31 to 48 years (n = 6, 30.00%), and 61 to 73 years (n = 2, 10.00%).

Table 1 provides the test–retest reliability and internal consistency measures for the Brazilian Portuguese version of the Cooking and Food Skill Confidence Questionnaire. The table is divided into two sections: one showing the results for two online questionnaire administrations (online–online) and the other comparing an online with a printed administration (online–printed). Intraclass correlation coefficients (ICCs) were calculated to assess reliability across both formats, with high ICC values (above 0.8) indicating strong reliability between versions. Cronbach’s Alpha was used to evaluate internal consistency, with values above 0.7 supporting the questionnaire’s reliability.

### 3.2. Nationwide Survey Application

#### 3.2.1. Participants’ Socio-Demographic Characteristics

A total of 1266 individuals accessed the survey, agreed to participate, and thoroughly answered the survey. As shown in Table 2, most participants were female (84.0%; n = 1063); the sample mean age (SD) was 40 ± 14.13 y/o. Most participants were at least undergraduates (84.7%; n = 1072) and had a monthly income between 2 and 20 minimum wages (75.6%; n = 958).

#### 3.2.2. Cooking and Food Skill Confidence of Brazilian Adult Population

From the original 1266 individuals, only those who rated their ability on a scale of one to seven were considered for analysis, excluding participants who indicated that they did not use the skill (option zero on the scale). In this sense, each item presents the number of responses analyzed, comprising 1266 minus the number of participants who marked themselves as “0”, to evaluate people’s confidence in using the skill. “Peel and chop vegetables” (n = 1254; 99.1%), “Chop, mix and stir foods” (n = 1253; 99.0%) and “Use herbs and spices” (n = 1241; 98.0%) were the three most used abilities mentioned by the participants in the cooking skills part of the instrument (Table 3). Considering the part related to food skills, “read the best-before date on food?” (n = 1259; 99.4%), “keep basic items in your cupboard for putting meals together?” (n = 1235; 97.6%), and “prepare or cook a meal with limited time?” (n = 1234; 97.5%) were the most mentioned.

Key findings include a higher confidence in basic tasks such as peeling and cutting vegetables (mean score: 6.0 ± 1.2) and preparing meat (mean score: 5.8 ± 1.3). More complex tasks like “preparing sauces from scratch” had lower confidence levels (mean score: 4.3 ± 1.5). This reflects varying confidence levels across different cooking skills, with basic tasks showing higher confidence than more complex ones.

In the food skill section, the most frequently reported abilities included “read the best-before date on food?” (n = 1259; 99.4%), “keep basic items in your cupboard for putting meals together?” (n = 1235; 97.6%), and “prepare or cook a meal with limited time?” (n = 1234; 97.5%) (Table 3). These findings highlight the participants’ engagement in fundamental food-related practices.

In terms of confidence, the participants expressed higher levels of assurance in tasks such as “shop with a grocery list” (mean score: 5.24 ± 2.03) and “plan how much food to buy” (mean score: 5.33 ± 1.85). Conversely, lower confidence levels were observed in tasks such as “follow recipes when cooking” (mean score: 3.63 ± 2.02) and “buy cheaper cuts of meat” (mean score: 3.82 ± 2.03). This variation reflects differing confidence levels across food skills, with basic tasks generally eliciting greater confidence than more complex activities.

##### Analysis of Cooking and Food Skill Scores by Sex, Age, and Educational Level

Table 4 presents an overview of the cooking and food skill scores, segregated by sex, age, and educational level. The overall cooking skills score is significantly higher for female participants than for male participants (*p* < 0.001). However, there are no notable differences in cooking skills between age groups or schooling. Regarding food skills, the data show minor differences between groups, with female participants scoring higher than male participants (*p* < 0.001). This analysis indicates that, while gender plays a role in influencing cooking and food skills, age and educational level do not significantly affect these competencies. In general, the participants presented a higher ability to prepare and serve food effectively and safely (CS) than the abilities and knowledge needed to prepare safe, nutritious, and culturally appropriate meals (FSs) (*p* < 0.001).

### 3.3. Eating Competence of Brazilian Adult Population

The data presented in Table 5 analyze EC among the participants, segregated by sex, age, and educational level. The EC score, measured using the ecSI2.0 scale, is divided into sub-scores, including eating attitude, food acceptance, internal regulation, and contextual skills. While the overall EC does not differ significantly between groups, variations are observed in specific sub-scores. For instance, female participants show significantly higher contextual skills (*p* = 0.000) than male participants. Additionally, older participants (over 40 years) exhibit stronger internal regulation and contextual skills than younger participants (*p* = 0.001 and *p* = 0.026, respectively).

### 3.4. Associations Between Cooking and Food Skills and Eating Competence in Brazilian Adults

Table 6 shows that all items within cooking skills demonstrated positive correlations with the domains of EC, food acceptance, internal regulation, contextual skills, and the total score of ecSI2.0™BR. The strongest correlation was observed between making sauces and gravies from scratch and the eating attitude domain (r = 0.313 to 0.356, *p* < 0.001) and the total EC score (r = 0.356). On the other hand, the weakest positive correlation was between microwaving food and the internal regulation domain (r = 0.016 to 0.088, *p* < 0.001), with minimal association with the total score for ecSI2.0™BR (r = 0.048). The overall cooking skill score displayed significant positive correlations across all domains, with a robust correlation with the total EC score (r = 0.417, *p* < 0.001).

Pearson’s correlation was used to assess the relationship between CFSs and EC. The statistical analysis revealed a significant correlation between food skills and the domains of eating attitudes, food acceptance, internal regulation, contextual skills, and the total score of ecSI2.0™BR. The overall CFS score showed significant correlations with all domains, with the strongest being contextual skills (r = 0.487, *p* < 0.001) and the overall score (r = 0.502, *p* < 0.001).

The analysis of Table 6 reveals that certain domains of EC are more strongly associated with CFSs than others. Specifically, food acceptance displays the most robust relationship with CSs. For example, in Table 6, the overall cooking skill score correlates r = 0.367 with food acceptance. In contrast, the domain of internal regulation shows relatively weaker correlations with cooking skills (r = 0.272 in Table 6). The domains of eating attitudes and contextual skills also show notable, though slightly weaker, correlations with CSs (r = 0.370 and r = 0.344, respectively, in Table 6).

As for FSs, contextual skills exhibit the highest correlation with food skills, with an overall score of r = 0.487, highlighting the significant role of environmental and situational factors in EC. In contrast, the connection between food skills and internal regulation is weaker, with a correlation of r = 0.334. Eating attitudes and food acceptance also show meaningful, though moderate, correlations, ranging from r = 0.401 to r = 0.404, indicating that, while food skills contribute to the overall EC, their impact varies across different EC domains.

Table 7 presents the association between cooking skills and ecSI2.0™BR (both categorized). According to this classification, 956 participants (75.5%) exhibited good cooking and food skills, and 674 participants (53.2%) were classified as competent. As also concluded in Table 7, individuals with cooking and food skills showed higher EC.

All scores (total and item-specific) for cooking and food skills, with few exceptions, showed a positive and significant correlation with all domains and the total score of the ecSI2.0™BR.

## 4. Discussion

A growing body of evidence highlights the critical role of cooking skills in promoting healthier dietary habits and overall well-being, making it essential to understand the factors influencing the development and maintenance of these skills within different populations. A study showed that consuming ready meals and dining out, common in a contemporary lifestyle, are associated with lower cooking and food skills and a higher weight status [1]. This shift toward convenience reflects current habits of prioritizing quick meal solutions, often at the expense of developing comprehensive cooking skills. A study showed that a lack of home cooking is linked to a poorer diet quality, reinforcing the need for interventions which revitalize cooking practices in everyday life [2].

The sample in this study shows a predominance of female participants (84%), which aligns with the existing literature on food-related behaviors. Cultural norms often place meal preparation responsibilities on women, leading to their greater involvement in cooking activities and higher self-reported proficiency in cooking skills [36,37]. Studies indicate that women are more likely to take on cooking roles, viewing meal preparation as part of family caregiving, which may explain their interest in participating in studies focused on these skills [5,13,16,36,37].

In the Brazilian context, traditional expectations further reinforce women’s role in household food management, contributing to their over-representation in research related to cooking and food skills [10,28]. Therefore, the greater representation of women in our sample reflects the sociocultural context surrounding food preparation in Brazilian households.

Gender, educational level, and family income often influence an individual’s cooking and food skills and relationship with meals. For example, Lavelle et al. (2017) found that individuals with higher education are more likely to report better cooking and food skills, contributing to healthier eating behaviors and food choices [12]. Lavelle et al. (2017) demonstrated that women tend to acquire cooking skills at a younger age than men and are more likely to maintain these skills throughout adulthood, corroborating the results found in our study [12]. While the study carried out in 2017 shows a correlation, it is not possible to prove a direct causal relationship of gender, educational level, and family income with CFSs, as other factors like cultural norms and early exposure to cooking practices may also influence cooking skills.

The findings of our study indicate significant differences in participants’ overall cooking skills and food skills based on gender. Female participants demonstrated higher mean scores in both overall cooking skills (5.69 ± 3.78) and overall food skills (4.96 ± 1.01) compared to male participants (5.16 ± 3.93 and 4.64 ± 1.28, respectively), with *p*-values < 0.001 for both measures. This aligns with previous research indicating that women typically possess more cooking and food skills than men, likely due to societal norms and expectations surrounding gender roles in domestic cooking [16,17].

Regarding age, our results show no significant differences in cooking skills and food skills between those aged up to 40 years and those over 40 years. This finding is somewhat surprising given that older adults may have had more opportunities to develop cooking skills over their lifetimes. However, it is consistent with Lavelle et al. (2017), who suggested that learning cooking skills can occur at various life stages, and differences may not be as pronounced as expected across different age groups [12].

Studies provide supporting evidence for the relationship between education, income, and cooking skills, but they do not definitively prove causality. Instead, they highlight strong associations between these factors, suggesting that higher education and income levels tend to correlate with improved cooking skills and healthier food behaviors. Moreover, this pattern suggests that gender may be crucial in cooking behavior and dietary habits, as enhanced cooking skills are often associated with healthier eating practices [15].

For instance, while the total EC score did not significantly differ between male and female participants, the subdomain analysis showed that women scored higher in contextual skills (*p* < 0.001 *). This is consistent with Queiroz et al. [28], who found that women, particularly in Brazil, often assume greater responsibility for meal preparation and household food management, enhancing their contextual skills. In contrast, Queiroz et al. reported that men often display a more relaxed attitude toward eating, a pattern supported by the higher scores that male participants achieved in internal regulation (*p* = 0.010), as shown in Table 5 [28].

Higher education levels are often associated with better overall health behaviors, including more competent food choices and a greater ability to navigate food environments. However, unlike in Queiroz et al.’s study, where education was a strong determinant of EC among Brazilian adults, this effect was less pronounced in our study (Table 5) but still suggested a positive relationship [28].

The results in Table 5 show that individuals over 40 y/o scored higher in internal regulation (*p* = 0.001) and contextual skills (*p* = 0.026) compared to younger adults. This aligns with Queiroz et al.’s findings, which suggest that older individuals are generally more experienced in managing their eating habits, likely because they have had more time to establish consistent eating patterns and are less likely to be influenced by modern dietary trends which emphasize convenience and processed foods [28].

The analysis in this research also revealed a direct correlation between higher levels of cooking and food skills and greater EC, highlighting that these skills are important for a healthier and more autonomous relationship with food. Individuals with more developed cooking skills can prepare balanced meals, plan their shopping and meals in advance, and avoid the excessive consumption of industrialized foods—elements which significantly contribute to the domains of internal regulation and contextual skills within EC, for example. Various studies can indirectly underscore the significance of cooking and food skills in promoting EC. Cooking skills are not merely about food preparation; they are crucial in shaping dietary habits and nutritional outcomes. For instance, Van Der Horst et al. (2011) highlighted that individuals with higher cooking skills tend to consume fewer ready meals, often associated with poorer nutritional profiles and a higher weight status. This finding suggests that enhanced cooking skills can lead to healthier food choices and better weight management [1].

This is reinforced by the work of McGowan et al. (2017), who reviewed the role of domestic cooking in food choices, finding that individuals with solid cooking skills tended to exhibit more balanced dietary patterns. Similarly, Hartmann et al. (2013) illustrated that cooking skills contribute to healthier food choices, which are essential components of EC [13,16].

One of the most robust findings from the present research is the direct positive correlation between higher levels of cooking skills and greater EC. Investing in educational initiatives to improve cooking skills is crucial. The growing dependence on processed and convenience foods, often due to a lack of time or skills, is a well-documented trend and can negatively impact EC [1]. A low EC is linked to a range of adverse health outcomes, including obesity, poor dietary quality, and an increased risk of chronic diseases such as diabetes and cardiovascular diseases [28,29]. Furthermore, Arslan et al. (2022) found that a lack of food and cooking skills contributes to disordered eating behaviors and poorer dietary patterns among individuals with overweight or obesity, highlighting the need for interventions to improve cooking skills and thus foster healthier eating habits [6].

The relationship between cooking and food skills and EC is multifaceted, profoundly influencing different aspects of individuals’ interactions with food and their overall dietary quality. As conceptualized by Satter (2007), EC comprises four key domains: eating attitudes, food acceptance, internal regulation, and contextual skills [38]. Each of these domains is intrinsically linked to cooking and food skills, and a deeper understanding of this relationship reveals how improved cooking abilities can lead to healthier eating behaviors and better health outcomes.

The domain of eating attitudes involves an individual’s perceptions, feelings, and behaviors towards food and eating, encompassing aspects such as enjoyment of eating, positive experiences with food, and confidence in managing eating situations. A positive attitude towards food encourages individuals to recognize the value of a well-balanced diet and the importance of investing time in selecting, preparing, and enjoying meals [11]. Hartmann et al. (2013) highlight that possessing cooking skills can significantly influence positive eating attitudes, as they provide individuals with the confidence to prepare diverse meals, thus fostering a more varied and enjoyable diet [16]. The ability to cook allows for greater control over ingredients and portion sizes, which may enhance meal satisfaction and promote a healthier approach to food consumption.

The results from our study indicate a moderate but meaningful correlation between food skills and the domain of eating attitudes. This suggests that food skills contribute to more positive attitudes toward eating, but they are not the sole factor. For example, cooking is considered a waste of time and a negative eating attitude [39]. This perspective reinforces that eating attitudes are multifaceted, influenced not only by food skills but also by cultural, experiential, and psychological factors [19,38].

Lavelle et al. (2016) note that the influence of food skills on eating attitudes might be moderated by these other psychological and cultural elements, which also shape how individuals perceive and interact with food [5]. In comparison, while food and cooking skills have an impact, cooking skills tend to show a slightly stronger correlation with eating attitudes, potentially due to the more hands-on nature of cooking and its role in fostering a deeper connection to the food preparation process.

Comparing the relationship between cooking skills and food skills with the eating attitude domain, it is evident that cooking skills showed a slightly stronger correlation (e.g., r = 0.370 and 0.401, respectively). In addition, Lavelle et al. (2017) demonstrated that higher food skills can foster a more proactive and positive approach to eating by enhancing individuals’ ability to manage food choices, experiment with ingredients, and prepare meals that are both satisfying and nutritious [12]. Furthermore, participants who reported greater confidence in fundamental food skills, such as reading food labels or preparing meals with limited ingredients, exhibited a more mindful and engaged attitude toward eating.

Food acceptance is particularly relevant in contexts where limited cooking skills may lead to reliance on processed or convenience foods, which tend to lack variety and nutrient density [1]. The present study found a strong correlation between cooking skills and the food acceptance EC domain (Table 6), with participants demonstrating a higher tendency to try new foods and embrace variety. These findings are consistent with Hartmann, Dohle, and Siegrist (2013)’s study, where they also observed that individuals with better cooking abilities were more likely to consume a wider variety of fruits, vegetables, and other nutrient-dense foods. Preparing and experimenting with fresh ingredients is essential for developing a diverse and balanced diet, a fundamental aspect of EC [15,16].

The stronger correlation between food skills and the food acceptance domain, as observed in Table 6, can be attributed to the specific nature of food skills, encompassing a broader range of behaviors related to meal planning, grocery shopping, and making deliberate food choices. These skills directly support the willingness to try new foods and incorporate a wider variety of ingredients into one’s diet, which are key components of food acceptance [12].

For instance, planning meals ahead, shopping with a grocery list, or keeping basic items in the cupboard for meal preparation encourages individuals to be more adventurous in their food choices, as they are better equipped to manage a diverse range of ingredients and plan balanced meals. This is because they feel more confident managing a variety of ingredients and planning balanced meals, which aligns with findings by Hartmann et al. (2013), who noted that individuals with better food skills tend to have more varied diets, including higher fruit and vegetable consumption [16].

In contrast, while cooking skills also play a role in food acceptance, their focus is more on the technical aspects of preparing food, such as chopping, mixing, or making sauces. These abilities might be essential for the practical execution of meal preparation but may not directly influence the willingness to try new foods to the same extent as food skills. Cooking skills are more concerned with the mechanical processes of transforming ingredients into meals, which, while important, do not necessarily encourage the exploration of new foods like food management and planning skills [12,16].

Although both skill sets are directly correlated with food acceptance, the direct connection between food skills and the ability to manage and plan meals might explain their more substantial influence on fostering dietary variety and openness.

Additionally, EC involves the internal regulation of eating, meaning the ability to recognize hunger and satiety cues and act accordingly [20]. Cooking skills can facilitate this self-regulation, as cooking fosters a greater connection with the eating process, making it more mindful. By preparing meals from scratch, individuals have more control over portions, ingredients, and preparation methods, which can help them make more appropriate choices regarding the amount of food consumed and the nutritional quality of meals [16,22].

Lohse et al. (2007) highlight that cooking skills contribute to a more mindful eating approach, helping individuals regulate their food intake based on internal signals rather than external cues such as portion sizes or convenience food availability. Cooking from scratch encourages individuals to connect more deeply with their meals, fostering greater self-regulation and appropriate eating behaviors [20].

The EC domain of contextual skills, which involves managing the food environment—such as meal planning, grocery shopping, and resource management—is also enhanced by cooking and food skills, but mainly the latter. This is particularly evident in the significant correlation between food skills and contextual skills observed in this study (r = 0.487, *p* < 0.001) (Table 6). This strong association indicates that individuals with advanced food skills, such as effectively planning meals and using leftovers, are better equipped to manage their food environment efficiently. Such skills allow them to shop strategically, minimize food waste, and make the most of available ingredients, thus supporting a more competent approach to eating.

A significant positive correlation was observed between overall food skills and EC, particularly in the domain of contextual skills, with a correlation coefficient of r = 0.487 (*p* < 0.001) (Table 6). This indicates that individuals who demonstrate proficiency in food skills, such as planning meals and utilizing leftovers, are better equipped to manage their food environment effectively. These findings align with research by Lavelle et al. (2017), which suggests that improved food skills are associated with better food management practices, including meal planning and reducing food waste [40].

In this study, there was a strong correlation between food skills and contextual skills (Table 4), with participants demonstrating greater efficiency in using leftovers, planning meals, and shopping with specific dishes in mind. These findings are aligned with research by Lavelle et al. (2017), which showed that individuals with better cooking and food skills were more likely to exhibit superior contextual management in food-related activities, such as budgeting for groceries and reducing food waste [12].

While basic food skills such as reading food labels (“reading the best-before date” and “reading the storage and use-by information on food packets”) were almost universally practiced among participants, more complex skills like “cooking more or doubling recipes for another meal” showed lower frequency and confidence levels [12].

Specifically, microwaving food showed the lowest correlation among all assessed skills (r = 0.048, *p* = 0.006) with the overall eating competence score (Table 6); it is an ability that does not require high cooking skills, so this might be the reason why this is not directly related to higher EC. As a result, it may not contribute as significantly to the development of broader competencies like meal planning, ingredient selection, or food preparation, which are more directly associated with higher levels of EC. This aligns with research by Arslan et al. (2023), highlighting that individuals who engage predominantly in basic food preparation practices often have lower scores in food-related behaviors [6].

The ability to “prepare or cook a healthy meal with only a few ingredients” was notably associated with improved EC, showing a strong correlation with the total score (r = 0.414, *p* < 0.001) (Table 6). This suggests that food skills that promote flexibility in meal preparation are crucial for fostering healthier eating behaviors, as individuals are more capable of adapting to different situations and making nutritious choices even when resources are limited [13]. These findings support the importance of developing food skills as a strategy for enhancing eating competence and dietary quality [11]

Furthermore, enhancing EC through improved cooking skills can have significant implications for public health, especially in a country as vast and socioeconomically diverse as Brazil. The positive relationship between cooking skills and EC indicates that equipping people with the ability to cook could foster a more food-competent population. This, in turn, could reduce the prevalence of non-communicable diseases such as obesity and type 2 diabetes, which are often linked to a poor dietary quality [7].

Finally, when examining the relationship between cooking skills and EC, it is essential to consider the potential positive impact of this correlation on public health policies. Improved cooking skills are associated with a greater ability to choose healthier foods, control portions, and enjoy a varied diet, factors which significantly contribute to the prevention of chronic non-communicable diseases such as obesity and type 2 diabetes [7].

Promoting cooking education can effectively improve public health and foster a healthier and more competent relationship with food. Thus, it is evident that cooking and food skills not only improve a diet’s quality but also impact EC, providing a solid foundation for healthier and more sustainable eating behaviors.

### Study Strengths and Potential Limitations

We used validated instruments for cooking and food skills and eating competence, including the Cooking and Food Skill Confidence Questionnaire (CFSCQ-BR) and the ecSI2.0™BR. These tools have been previously validated in various international contexts and adapted for the Brazilian population. The rigorous process of cultural adaptation and validation ensures that the data collected accurately reflect the participants’ competencies and behaviors concerning food preparation and consumption. This increases the study’s methodological credibility and supports the reliability of the results.

Furthermore, using a cross-sectional design combined with robust statistical analysis techniques, such as Pearson’s and Spearman’s correlation tests, allowed for a detailed examination of the association between cooking skills and eating competence. The significant correlations across multiple domains, including food attitudes, internal regulation, and contextual skills, provide a comprehensive understanding of the multidimensional relationship between cooking skills and eating competence.

Additionally, the accessibility of the online survey format facilitated widespread participation across a large and diverse demographic, reducing recruitment costs and time while allowing for a larger sample size. This approach effectively reached participants from various regions, which is often a challenge in a country of Brazil’s size.

This study has limitations. The sample, while large, was collected using a non-probabilistic convenience sampling method. This might have limited the generalizability of the findings to the broader Brazilian population. Moreover, most participants were female (83.81%), a common trend in studies involving food and nutrition behaviors, as women are often more engaged in food preparation activities [17].

The over-representation of female participants in this study reflects a broader societal pattern where women are more likely to engage in cooking activities. Several studies suggest that cultural and social expectations often place the responsibility of cooking and food preparation on women, which may explain their greater interest and involvement in studies on cooking skills [17]. Women also tend to perceive cooking as care for their families, which could contribute to their higher participation rates in research focused on food and cooking behaviors [12].

The under-representation of male participants could influence the results, given that men typically report lower cooking skills [12]. Future research should aim for a more balanced gender representation to provide a more comprehensive understanding of the population’s cooking skills and eating competence.

Geographical limitations are also present, as the study used an online recruitment method which may not have reached participants in rural or remote areas of Brazil. The vast size and diversity of the country make it difficult to capture a fully representative sample, which is a significant challenge for nationwide studies in Brazil.

## 5. Conclusions

This is the first study evaluating the correlation between CFSs and EC. Our findings show that individuals with higher confidence in their cooking abilities enjoy a greater diet variety, improved self-regulation in eating, and better adaptability to their food environments. These results highlight the potential for public health interventions to improve EC by fostering cooking skills, which could lead to healthier dietary behaviors, and, ultimately, improved public health outcomes.

Given the critical role of these skills in health promotion, similar research in various cultural and socioeconomic contexts could provide broader insights into how cooking skills affect EC worldwide. Studies in countries where the Eating Competence Inventory has been validated—such as the United States, Italy, and Germany—could help determine whether these associations hold across diverse populations. Addressing gaps in gender representation and geographic diversity would enhance future studies and enable targeted, culturally relevant interventions.

Future research should aim to include more diverse and representative samples, particularly concerning gender and geography, to explore these associations further. Public health initiatives focused on cooking skills education could be instrumental in fostering a healthier, more eating-competent population in Brazil.

Furthermore, expanding this research to include various populations, including low-income groups, children, and older adults, could develop targeted educational programs that promote cooking skills and, consequently, enhance eating competence across different cultural backgrounds.

## Figures and Tables

**Table 1:** Statistical analysis of the validation data for the Cooking and Food Skill Confidence Questionnaire (Brazilian Portuguese version) across online–online and online–printed formats.

	Mean (SD) *	Cronbach’s Alpha	ICC (95% CI) **
Online–Online			
Online 1	143.45 (39.92)	0.928	0.906 (0.768; 0.963)
Online 2	148.75 (31.74)	0.887	
Online–Printed			
Online	142.30 (54.55)	0.944	0.887 (0.719; 0.955)
Printed	148.50 (44.94)	0.971	

* Cooking and Food Skill Confidence Questionnaire mean score. ** ICC: intraclass correlation coefficient; and CI: confidence interval.

**Table 2:** Participants’ socio-demographic characteristics, Brazil, 2024 (n = 1266).

		Sample (n = 1266)	
		N	%
Gender	Female	1063	84.0%
	Male	203	16.0%
Age	Up to 30 years	407	32.1%
	31 to 40 years	305	24.1%
	41 to 50 years	252	19.9%
	51 years to 60 years	164	13.0%
	61 years or older	138	10.9%
Schooling Level	Up to High school	194	15.3%
	Undergraduate	786	62.1%
	Graduate	286	22.6%
Income *	Up to 1 MW	43	3.4%
	>1 MW to 2 MW	89	7.0%
	>2 MW to 5 MW	314	24.8%
	>5 MW to 10 MW	346	27.3%
	>10 MW to 20 MW	298	23.5%
	More than 20 MW	117	9.2%
	Prefer not to report or Do not know	59	4.7%

* 1 MW = 258.74 USD (3 October 2024).

**Table 3:** Participants’ cooking and food skill measures, Brazil, 2024.

	Usage	Confidence (Rated 1–7)
	N (%)	Mean (SD)
Cooking Skills		
1. Chop, mix, and stir foods	1253 (99.0%)	6.04 (1.31)
2. Blend foods to make them smooth, like soups or sauces	1216 (96.1%)	6.10 (1.32)
3. Steam food	1142 (90.2%)	5.85 (1.49)
4. Boil or simmer food	1232 (97.3%)	5.92 (1.36)
5. Stew food	1167 (92.2%)	5.32 (1.66)
6. Roast food in the oven	1204 (95.1%)	5.75 (1.39)
7. Fry/stir-fry food in a frying pan/wok with oil or fat	1200 (94.8%)	5.50 (1.67)
8. Microwave food	1078 (85.2%)	5.37 (1.71)
9. Bake goods	1106 (87.4%)	5.07 (1.78)
10. Peel and chop vegetables	1254 (99.1%)	6.01 (1.39)
11. Prepare and cook raw meat/poultry	1174 (92.7%)	5.86 (1.44)
12. Prepare and cook raw fish	1062 (83.9%)	5.05 (1.77)
13. Make sauces and gravy from scratch	1108 (87.5%)	5.15 (1.75)
14. Use herbs and spices	1241 (98.0%)	5.92 (1.46)
Overall cooking skill score		5.60 (1.07)
Food Skills		
1. Plan meals ahead?	1175 (92.8%)	4.20 (2.09)
2. Prepare meals in advance?	1117 (88.2%)	4.03 (2.14)
3. Follow recipes when cooking?	1199 (94.7%)	3.63 (2.02)
4. Shop with a grocery list?	1202 (94.9%)	5.24 (2.03)
5. Shop with specific meals in mind?	1202 (94.9%)	4.58 (1.95)
6. Plan how much food to buy?	1216 (96.1%)	5.33 (1.85)
7. Compare prices before you buy food?	1204 (95.1%)	5.03 (2.13)
8. Know what budget you have to spend on food?	1159 (91.5%)	5.05 (2.03)
9. Buy food in season to save money?	1164 (91.9%)	4.79 (2.06)
10. Buy cheaper cuts of meat to save money?	1004 (79.3%)	3.82 (2.03)
11. Cook more or double recipes which can be used for another meal?	1183 (93.4%)	5.05 (1.87)
12. Prepare or cook a healthy meal with only few ingredients on hand?	1226 (96.8%)	5.24 (1.61)
13. Prepare or cook a meal with limited time?	1234 (97.5%)	5.20 (1.64)
14. Use leftovers to create another meal?	1206 (95.3%)	4.97 (2.03)
15. Keep basic items in your cupboard for putting meals together?	1235 (97.6%)	5.86 (1.62)
16. Read the best-before date on food?	1259 (99.4%)	6.16 (1.51)
17. Read the storage and use-by information on food packets?	1214 (95.9%)	5.02 (2.08)
18. Read the nutrition information on food labels?	1200 (94.8%)	5.04 (2.09)
19. Balance meals based on nutrition advice on what is healthy?	1197 (94.5%)	5.19 (1.79)
Overall food skill score		4.91 (0.92)

**Table 4:** Overall scores of cooking and food skill scale segregated by sex, age, and educational level, Brazil, 2024 (n = 1266).

	Overall Cooking Skill	Overall Food Skill	*p* ^1^
	Mean (SD)	Mean (SD)	
Total (n = 1266)	5.60 (1.07)	4.91 (0.92)	<0.001 *
Gender			
Female (n = 1063)	5.69 (3.78) ^B^	4.96 (1.01) ^B^	<0.001 *
Male (n = 203)	5.16 (3.93) ^A^	4.64 (1.28) ^A^	<0.001 *
*p* ^2^	<0.001 **	<0.001 **	<0.001 *
Age			
Up to 40 years (n = 712)	5.59 (1.03) ^A^	4.91 (0.87) ^A^	<0.001 *
More than 40 years (n = 554)	5.62 (1.13) ^A^	4.94 (0.97) ^A^	<0.001 *
*p* ^2^	0.714 **	0.991 **	<0.001 *
Educational level			
High school (n = 194)	5.66 (0.98) ^A^	4.82 (0.96) ^A^	<0.001 *
Undergraduate (n = 786)	5.58 (1.06) ^A^	4.91 (0.90) ^A^	<0.001 *
Graduate (n = 286)	5.63 (1.16) ^A^	4.96 (0.96) ^A^	<0.001 *
*p* ^2^	0.561 ***	0.302 ***	<0.001 *

^1^ Test comparing cooking and food skills; ^2^ test comparing cooking and food skill scores with variables of interest; * paired Student *t*-test; ** independent Student *t*-test; and *** ANOVA with Tukey’s post hoc test. Groups with the same letters in the columns do not differ significantly.

**Table 5:** Sub-scores and categories of the ecSI2.0 scale segregated by sex, age, and educational level, Brazil (n = 1266).

	Eating Attitude	Food Acceptance	Internal Regulation	Contextual Skills	Total	ecSI2.0 ≥ 32
	Mean (SD)	Mean (SD)	Mean (SD)	Mean (SD)	Mean (SD)	Freq. (%)
Gender						
Female (n = 1063)	12.51 (3.78) ^A^	5.62 (2.22) ^A^	3.60 (1.74) ^A^	9.94 (3.22) ^B^	31.66 (9.12) ^A^	577 (54.3%) ^A^
Male (n = 203)	12.37 (3.93) ^A^	5.73 (2.25) ^A^	3.38 (1.79) ^A^	8.98 (3.51) ^A^	30.46 (9.42) ^A^	97 (47.8%) ^A^
*p*	0.639 *	0.507 *	0.103 *	<0.001 *	0.086 *	0.089 ***
Age						
Up to 40 years (n = 712)	12.55 (3.73) ^A^	5.71 (2.26) ^A^	3.42 (1.70) ^A^	9.60 (3.26) ^A^	31.29 (8.99) ^A^	375 (52.7%) ^A^
More than 40 years (n = 554)	12.40 (3.91) ^A^	5.53 (2.17) ^A^	3.76 (1.78) ^B^	10.02 (3.31) ^B^	31.71 (9.40) ^A^	299 (54.0%) ^A^
*p*	0.464 *	0.150 *	0.001 *	0.026 *	0.420 *	0.645 ***
Educational level						
High school (n = 194)	12.47 (3.73) ^A^	5.57 (2.30) ^A^	3.45 (1.79) ^A^	9.36 (3.31) ^A^	30.86 (8.91) ^A^	96 (49.57%) ^A^
Undergraduate (n = 786)	12.44 (3.87) ^A^	5.59 (2.23) ^A^	3.60 (1.73) ^A^	9.78 (3.28) ^A^	31.41 (9.27) ^A^	421 (53.6%) ^A^
Graduate (n = 286)	12.62 (3.68) ^A^	5.79 (2.14) ^A^	3.55 (1.75) ^A^	10.08 (3.28) ^A^	32.04 (9.07) ^A^	157 (54.9%) ^A^
*p*	0.798 **	0.384 **	0.523 **	0.061 **	0.369 **	0.485 ***

* Student *t*-test; ** ANOVA with Tukey’s post hoc test; and *** Pearson’s Chi-square test. Groups with the same letters do not differ significantly.

**Table 6:** Association between cooking and food skills and ecSI2.0™BR, Brazil.

			ecSI2.0™BR		
	Eating Attitudes	Food Acceptance	Internal Regulation	Contextual Skills	Total
	Corr (*p*)	Corr (*p*)	Corr (*p*)	Corr (*p*)	Corr (*p*)
1. Chop, mix and stir foods	0.300 (<0.001 *) *	0.266 (<0.001 *) *	0.227 (<0.001 *) *	0.254 (<0.001 *) *	0.324 (<0.001 *) *
2. Blend foods to make them smooth, like soups or sauces	0.255 (<0.001 *) *	0.246 (<0.001 *) *	0.178 (<0.001 *) *	0.237 (<0.001 *) *	0.286 (<0.001 *) *
3. Steam food	0.235 (<0.001 *) *	0.229 (<0.001 *) *	0.220 (<0.001 *) *	0.253 (<0.001 *) *	0.286 (<0.001 *) *
4. Boil or simmer food	0.272 (<0.001 *) *	0.271 (<0.001 *) *	0.226 (<0.001 *) *	0.299 (<0.001 *) *	0.328 (<0.001 *) *
5. Stew food	0.285 (<0.001 *) *	0.272 (<0.001 *) *	0.230 (<0.001 *) *	0.277 (<0.001 *) *	0.327 (<0.001 *) *
6. Roast food in the oven	0.264 (<0.001 *) *	0.256 (<0.001 *) *	0.229 (<0.001 *) *	0.277 (<0.001 *) *	0.317 (<0.001 *) *
7. Fry/stir-fry food in a frying pan/wok with oil or fat	0.194 (<0.001 *) *	0.207 (<0.001 *) *	0.123 (<0.001 *) *	0.168 (<0.001 *) *	0.214 (<0.001 *) *
8. Microwave food	0.061 (0.044) *	0.088 (0.004) *	0.016 (0.608) *	0.087 (0.004) *	0.048 (0.006) *
9. Bake goods	0.210 (<0.001 *) *	0.196 (<0.001 *) *	0.162 (<0.001 *) *	0.227 (<0.001 *) *	0.244 (<0.001 *) *
10. Peel and chop vegetables	0.302 (<0.001 *) *	0.299 (<0.001 *) *	0.249 (<0.001 *) *	0.295 (<0.001 *) *	0.348 (<0.001 *) *
11. Prepare and cook raw meat/poultry	0.269 (<0.001 *) *	0.266 (<0.001 *) *	0.199 (<0.001 *) *	0.194 (<0.001 *) *	0.279 (<0.001 *) *
12. Prepare and cook raw fish	0.274 (<0.001 *) *	0.280 (<0.001 *) *	0.254 (<0.001 *) *	0.211 (<0.001 *) *	0.300 (<0.001 *) *
13. Make sauces and gravy from scratch	0.313 (<0.001 *) *	0.290 (<0.001 *) *	0.263 (<0.001 *) *	0.299 (<0.001 *) *	0.356 (<0.001 *) *
14. Use herbs and spices	0.304 (<0.001 *) *	0.261 (<0.001 *) *	0.274 (<0.001 *) *	0.288 (<0.001 *) *	0.345 (<0.001 *) *
Overall cooking skills score	0.370 (<0.001 *) **	0.367 (<0.001 *) **	0.272 (<0.001 *) **	0.344 (<0.001 *) **	0.417 (<0.001 *) **
Food Skills					
1. Plan meals ahead?	0.203 (<0.001 *) *	0.236 (<0.001 *) *	0.192 (<0.001 *) *	0.323 (<0.001 *) *	0.296 (<0.001 *) *
2. Prepare meals in advance?	0.188 (<0.001 *) *	0.229 (<0.001 *) *	0.106 (<0.001 *) *	0.258 (<0.001 *) *	0.250 (<0.001 *) *
3. Follow recipes when cooking?	0.038 (0.193)*	0.025 (0.388)*	−0.017 (0.565)*	0.013 (0.664)*	0.021 (0.477)*
4. Shop with a grocery list?	0.169 (<0.001 *) *	0.140 (<0.001 *) *	0.135 (<0.001 *) *	0.200 (<0.001 *) *	0.202 (<0.001 *) *
5. Shop with specific meals in mind?	0.178 (<0.001 *) *	0.169 (<0.001 *) *	0.133 (<0.001 *) *	0.211 (<0.001 *) *	0.218 (<0.001 *) *
6. Plan how much food to buy?	0.308 (<0.001 *) *	0.227 (<0.001 *) *	0.287 (<0.001 *) *	0.334 (<0.001 *) *	0.357 (<0.001 *) *
7. Compare prices before you buy food?	0.157 (<0.001 *) *	0.141 (<0.001 *) *	0.158 (<0.001 *) *	0.178 (<0.001 *) *	0.189 (<0.001 *) *
8. Know what budget you have to spend on food?	0.227 (<0.001 *) *	0.156 (<0.001 *) *	0.217 (<0.001 *) *	0.212 (<0.001 *) *	0.243 (<0.001 *) *
9. Buy food in season to save money?	0.245 (<0.001 *) *	0.200 (<0.001 *) *	0.244 (<0.001 *) *	0.308 (<0.001 *) *	0.304 (<0.001 *) *
10. Buy cheaper cuts of meat to save money?	0.087 (0.006)*	0.087 (0.006)*	0.019 (0.540)*	0.018 (0.561)*	0.067 (0.033)*
11. Cook more or double recipes which can be used for another meal?	0.078 (0.007)*	0.114 (<0.001 *) *	0.029 (0.540)*	0.159 (<0.001 *) *	0.120 (<0.001 *) *
12. Prepare or cook a healthy meal with only few ingredients on hand?	0.325 (<0.001 *) *	0.352 (<0.001 *) *	0.300 (<0.001 *) *	0.381 (<0.001 *) *	0.414 (<0.001 *) *
13. Prepare or cook a meal with limited time?	0.296 (<0.001 *) *	0.294 (<0.001 *) *	0.209 (<0.001 *) *	0.262 (<0.001 *) *	0.325 (<0.001 *) *
14. Use leftovers to create another meal?	0.196 (<0.001 *) *	0.266 (<0.001 *) *	0.155 (<0.001 *) *	0.207 (<0.001 *) *	0.249 (<0.001 *) *
15. Keep basic items in your cupboard for putting meals together?	0.215 (<0.001 *) *	0.252 (<0.001 *) *	0.151 (<0.001 *) *	0.276 (<0.001 *) *	0.274 (<0.001 *) *
16. Read the best-before date on food?	0.180 (<0.001 *) *	0.136 (<0.001 *) *	0.216 (<0.001 *) *	0.187 (<0.001 *) *	0.212 (<0.001 *) *
17. Read the storage and use-by information on food packets?	0.233 (<0.001 *) *	0.198 (<0.001 *) *	0.256 (<0.001 *) *	0.259 (<0.001 *) *	0.283 (<0.001 *) *
18. Read the nutrition information on food labels?	0.197 (<0.001 *) *	0.195 (<0.001 *) *	0.170 (<0.001 *) *	0.236 (<0.001 *) *	0.239 (<0.001 *) *
19. Balance meals based on nutrition advice on what is healthy?	0.335 (<0.001 *) *	0.311 (<0.001 *) *	0.336 (<0.001 *) *	0.402 (<0.001 *) *	0.421 (<0.001 *) *
Overall cooking skills score	0.401 (<0.001 *) **	0.404 (<0.001 *) **	0.334 (<0.001 *) **	0.487 (<0.001 *) **	0.502 (<0.001 *) **

* Spearman’s correlation coefficient; and ** Pearson’s correlation coefficient.

**Table 7:** Association between cooking and food skill total score and ecSI2.0™BR results in the Brazilian adult population (n = 1266).

		ecSI2.0™BR		Total	*p* *
		<32	≥32		
Cooking and Food Skills	<5	220 (71.0%)	90 (29.0%)	310	<0.001
	≥5	372 (38.9%)	584 (61.1%)	956	
Total		592	674	1266	

* Pearson’s Chi-squared test.

## Data Availability

The raw data supporting the conclusions of this article will be made available by the authors upon request. The data are not publicly available due to privacy and ethical reasons.

## Supplementary Materials

The following supporting information can be downloaded at https://www.mdpi.com/article/10.3390/nu16233980/s1: Table S1: Original English version of Cooking and Food Skill Confidence Questionnaire; Table S2: Brazilian Portuguese version of the Cooking and Food Skill Confidence Questionnaire; and Table S3: Gender and age distribution of participants in Groups 1 and 2 for the validation of the Brazilian Portuguese version of the Cooking and Food Skill Confidence Questionnaire.
