# Supplementary material for: Exploring the Relationship Between Cooking and Food Skills and Eating Competence Among Brazilian Adults
Source: Nutrients. 2024 Nov 21;16(23):3980. doi: 10.3390/nu16233980 (PMC11643768; doi:10.3390/nu16233980)
Supplement: Supplementary file 1 [file nutrients-16-03980-s001.zip › nutrients-3324522-supplementary.pdf]

SUPPLEMENTARY MATERIAL

Table S1. Original English version of Cooking and Food Skills Questionnaire.

**Cooking Skills**	
Item	Scale options
How good you are at:	
Cooking Method	
1. Chop, mix and stir foods	0. I do not do; 1. Very poor; 2; 3; 4; 5; 6; 7. Very good
2. Blend foods to make them smooth, like soups or sauces	0. I do not do; 1. Very poor; 2; 3; 4; 5; 6; 7. Very good
3. Steam food	0. I do not do; 1. Very poor; 2; 3; 4; 5; 6; 7. Very good
4. Boil or simmer food	0. I do not do; 1. Very poor; 2; 3; 4; 5; 6; 7. Very good
5. Stew food	0. I do not do; 1. Very poor; 2; 3; 4; 5; 6; 7. Very good
6. Roast food in the oven	0. I do not do; 1. Very poor; 2; 3; 4; 5; 6; 7. Very good
7. Fry/stir-fry food in a frying pan/wok with oil or fat	0. I do not do; 1. Very poor; 2; 3; 4; 5; 6; 7. Very good
8. Microwave food	0. I do not do; 1. Very poor; 2; 3; 4; 5; 6; 7. Very good
Food Preparation Techniques	
9. Bake goods	0. I do not do; 1. Very poor; 2; 3; 4; 5; 6; 7. Very good
10. Peel and chop vegetables	0. I do not do; 1. Very poor; 2; 3; 4; 5; 6; 7. Very good
11. Prepare and cook raw meat/poultry	0. I do not do; 1. Very poor; 2; 3; 4; 5; 6; 7. Very good
12. Prepare and cook raw fish	0. I do not do; 1. Very poor; 2; 3; 4; 5; 6; 7. Very good
13. Make sauces and gravy from scratch	0. I do not do; 1. Very poor; 2; 3; 4; 5; 6; 7. Very good
14. Use herbs and spices	0. I do not do; 1. Very poor; 2; 3; 4; 5; 6; 7. Very good
**Food Skills**	Scale options
How often do you:	
Meal Planning and Preparing	
1.Plan meals ahead?	0. I do not do; 1. Rarely; 2; 3; 4; 5; 6; 7. Always
2…prepare meals in advance?	0. I do not do; 1. Rarely; 2; 3; 4; 5; 6; 7. Always
3…follow recipes when cooking?	0. I do not do; 1. Rarely; 2; 3; 4; 5; 6; 7. Always
Shopping	
4…shop with a grocery list?	0. I do not do; 1. Rarely; 2; 3; 4; 5; 6; 7. Always
5…shop with specific meals in mind?	0. I do not do; 1. Rarely; 2; 3; 4; 5; 6; 7. Always
6…plan how much food to buy?	0. I do not do; 1. Rarely; 2; 3; 4; 5; 6; 7. Always
Budgeting	

7…compare prices before you buy food?	0. I do not do; 1. Rarely; 2; 3; 4; 5; 6; 7. Always
8…know what budget you have to spend on food?	0. I do not do; 1. Rarely; 2; 3; 4; 5; 6; 7. Always
9…buy food in season to save money?	0. I do not do; 1. Rarely; 2; 3; 4; 5; 6; 7. Always
10…buy cheaper cuts of meat to save money?	0. I do not do; 1. Rarely; 2; 3; 4; 5; 6; 7. Always
Resourcefulness	
11…cook more or double recipes which can be used for another meal?	0. I do not do; 1. Rarely; 2; 3; 4; 5; 6; 7. Always
12…prepare or cook a healthy meal with only few ingredients on hand?	0. I do not do; 1. Rarely; 2; 3; 4; 5; 6; 7. Always
13…prepare or cook a meal with limited time?	0. I do not do; 1. Rarely; 2; 3; 4; 5; 6; 7. Always
14…use leftovers to create another meal?	0. I do not do; 1. Rarely; 2; 3; 4; 5; 6; 7. Always
15...keep basic items in your cupboard for putting meals together?	0. I do not do; 1. Rarely; 2; 3; 4; 5; 6; 7. Always
Label reading/consumer awareness	
16…read the best-before date on food?	0. I do not do; 1. Rarely; 2; 3; 4; 5; 6; 7. Always
17…read the storage and use-by information on food packets?	0. I do not do; 1. Rarely; 2; 3; 4; 5; 6; 7. Always
18…read the nutrition information on food labels?	0. I do not do; 1. Rarely; 2; 3; 4; 5; 6; 7. Always
19…balance meals based on nutrition advice on what is healthy?	0. I do not do; 1. Rarely; 2; 3; 4; 5; 6; 7. Always

Table S2. Brazilian Portuguese version of Cooking and Food Skills Questionnaire.

**Habilidades Culinárias**	
Item	Opções de resposta
Em uma escala de 1 a 7, por favor responda quão bom você é em:	
Métodos culinários	
1.Picar, misturar e mexer alimentos. Exemplo: cortar vegetais, cortar uma cebola em cubos, cortar carne em cubos, misturar e mexer os alimentos em uma panela ou tigela	0. Não faço; 1. Muito ruim; 2; 3; 4; 5; 6; 7. Muito bom
2.Misturar/bater os alimentos para que fiquem mais cremosos, como sopas ou molhos (usando um batedor, liquidificador, processador de alimentos)	0. Não faço; 1. Muito ruim; 2; 3; 4; 5; 6; 7. Muito bom
3.Cozinhar alimentos no vapor (onde a comida não toca a água, mas são cozidos pelo vapor).	0. Não faço; 1. Muito ruim; 2; 3; 4; 5; 6; 7. Muito bom
4.Ferver ou cozinhar em fogo brando, ou seja, temperatura um pouco abaixo do ponto de fervura (cozinhar em uma panela com água quente, fervendo/borbulhando).	0. Não faço; 1. Muito ruim; 2; 3; 4; 5; 6; 7. Muito bom
5.Cozinhar em fogo lento - tempo de cozimento longo (geralmente mais de uma hora) em um líquido ou molho em fogo médio, sem ferver, por exemplo, ensopado/carne de panela.	0. Não faço; 1. Muito ruim; 2; 3; 4; 5; 6; 7. Muito bom
6.Cozinhar alimentos no forno, por exemplo, carne/frango, peixes, vegetais, etc.	0. Não faço; 1. Muito ruim; 2; 3; 4; 5; 6; 7. Muito bom
7.Fritar/refogar alimentos em uma frigideira ou outra panela, utilizando gordura e usando o fogão a gás ou elétrico.	0. Não faço; 1. Muito ruim; 2; 3; 4; 5; 6; 7. Muito bom
8.Preparar alimentos no microondas (considerando apenas refeições sólidas, excluindo bebidas e outros líquidos), inclusive aquecer refeições prontas.	0. Não faço; 1. Muito ruim; 2; 3; 4; 5; 6; 7. Muito bom

Técnicas de preparo dos alimentos	
9.Assar produtos de confeitaria como bolos, pãezinhos/pães, cupcakes, broas, rosquinha, bolacha/biscoito etc, usando ingredientes básicos/frescos ou misturas prontas de pacotes.	0. Não faço; 1. Muito ruim; 2; 3; 4; 5; 6; 7. Muito bom
10.Descascar e picar vegetais (como batatas, cenouras, cebolas e brócolis).	0. Não faço; 1. Muito ruim; 2; 3; 4; 5; 6; 7. Muito bom
11.Preparar e cozinhar carne bovina e de frango.	0. Não faço; 1. Muito ruim; 2; 3; 4; 5; 6; 7. Muito bom
12.Preparar e cozinhar peixes.	0. Não faço; 1. Muito ruim; 2; 3; 4; 5; 6; 7. Muito bom
13.Preparar molhos/cremes desde o início (do zero, sem misturas prontas em caixinha/lata/vidro).	0. Não faço; 1. Muito ruim; 2; 3; 4; 5; 6; 7. Muito bom
14.Usar ervas e temperos para dar sabor aos pratos.	0. Não faço; 1. Muito ruim; 2; 3; 4; 5; 6; 7. Muito bom
**Habilidades Alimentares**	
Em uma escala de 1 a 7, por favor responda com qual frequência você:	Scale options
Planejamento e preparo de refeições	
15…planeja refeições com antecedência? (por exemplo, para o dia/semana seguinte).	0. Não faço; 1. Raramente; 2; 3; 4; 5; 6; 7. Sempre
16... prepara refeições com antecedência? por exemplo, deixa alimentos já cortados e/ou temperado ou prepara parte dos pratos com antecedência para na hora ser mais rápido	0. Não faço; 1. Raramente; 2; 3; 4; 5; 6; 7. Sempre
17…segue receitas enquanto cozinha?	0. Não faço; 1. Raramente; 2; 3; 4; 5; 6; 7. Sempre
Compras	
18… faz compras com lista de produtos?	0. Não faço; 1. Raramente; 2; 3; 4; 5; 6; 7. Sempre
19…faz compras com refeições específicas em mente?	0. Não faço; 1. Raramente; 2; 3; 4; 5; 6; 7. Sempre
20…planeja a quantidade de alimento a ser comprada?	0. Não faço; 1. Raramente; 2; 3; 4; 5; 6; 7. Sempre
Orçamento	
21… compara preços antes de comprar alimentos?	0. Não faço; 1. Raramente; 2; 3; 4; 5; 6; 7. Sempre
22…sabe o quanto de dinheiro tem no orçamento para gastar em alimentos?	0. Não faço; 1. Raramente; 2; 3; 4; 5; 6; 7. Sempre
23… compra alimentos da época para economizar dinheiro?	0. Não faço; 1. Raramente; 2; 3; 4; 5; 6; 7. Sempre
24…compra cortes inferiores de carne para economizar? (Ex. optar por carnes de valor menor, independente da qualidade do insumo)	0. Não faço; 1. Raramente; 2; 3; 4; 5; 6; 7. Sempre
Desenvoltura	
25...cozinha a mais ou dobra receitas para comê-las em outra refeição?	0. Não faço; 1. Raramente; 2; 3; 4; 5; 6; 7. Sempre
26...prepara ou cozinha uma refeição saudável com apenas poucos ingredientes disponíveis?	0. Não faço; 1. Raramente; 2; 3; 4; 5; 6; 7. Sempre
27… prepara ou cozinha rapidamente uma refeição?	0. Não faço; 1. Raramente; 2; 3; 4; 5; 6; 7. Sempre
28… usa sobras para criar outra refeição?	0. Não faço; 1. Raramente; 2; 3; 4; 5; 6; 7. Sempre
29... mantem itens básicos no seu armário para preparar as refeições? Por exemplo ervas/especiarias, produtos desidratados/enlatados?	0. Não faço; 1. Raramente; 2; 3; 4; 5; 6; 7. Sempre
Leitura de rótulos/Consciência do consumidor	
30… lê a data de validade dos alimentos?	0. Não faço; 1. Raramente; 2; 3; 4; 5; 6; 7. Sempre

31…lê as informações do pacote sobre armazenamento e uso?	0. Não faço; 1. Raramente; 2; 3; 4; 5; 6; 7. Sempre
32… lê as informações nutricionais nos rótulos dos alimentos?	0. Não faço; 1. Raramente; 2; 3; 4; 5; 6; 7. Sempre
33...equilibra as refeições com base em aconselhamento nutricional sobre o que é saudável?	0. Não faço; 1. Raramente; 2; 3; 4; 5; 6; 7. Sempre

Table S3. Gender and age distribution of participants in Groups 1 and 2 for the validation of the Brazilian Portuguese version of the Cooking and Food Skills Questionnaire.

		Group 1 - Sample (n=21)		Group 2 - Sample (n=20)	
		N	%	N	%
Gender	Male	6	28.57%	9	45.00%
	Female	15	71.43%	11	55.00%
Age	21 to 30	11	52.38%	12	57.14%
	31 to 48	7	33.33%	6	28.57%
	61 to 73	3	14.29%	2	9.52%
